# Hypothyroidism Presenting Atypically as an Isolated Pericardial and Pleural Effusion: A Case Report

**DOI:** 10.7759/cureus.59255

**Published:** 2024-04-29

**Authors:** Samer Shaja, Mohammed A Khaleeluddin

**Affiliations:** 1 Family Medicine, JenCare Senior Medical Center, Glenwood, USA

**Keywords:** thyroid-stimulating hormone, atrial fibrillation, thyrotropin, bradycardia, levothyroxine, secondary hypothyroidism, pericardial effusion, pleural effusion, hypothyroidism

## Abstract

Hypothyroidism is an endocrine disorder characterized by low thyroid hormone levels, which commonly presents as fatigue, cold intolerance, constipation, poor memory and/or concentration, and weight gain. Common signs of hypothyroidism include bradycardia, electrocardiograph changes, a lower basal temperature, a slower relaxation phase of deep tendon reflexes, and swelling of the extremities. Hypothyroidism is diagnosed with labs showing high thyroid-stimulating hormone levels and low free thyroxine. Hypothyroidism may present as a pericardial or pleural effusion, with the incidence of each being unknown. The paucity of information regarding the incidence of pericardial and pleural effusions in hypothyroidism may be due to effusions being an atypical complication of a common endocrine disorder. Hypothyroidism, including in cases of pericardial or pleural effusions, is typically treated with thyroid hormone replacement therapy, usually in the form of levothyroxine. Hemodynamic compromise may necessitate pericardiocentesis or pleurocentesis. In this case report, we present an atypical presentation of hypothyroidism that is characterized by an isolated pericardial and pleural effusion in a patient with post-thyroidectomy hypothyroidism who was non-adherent to levothyroxine. We discuss the pathophysiology of pleural and pericardial effusions in thyroid disease, which is thought to involve increased capillary permeability and changes in oncotic pressure related to albumin. We also review treatment strategies regarding pericardial and pleural effusions in hypothyroidism.

## Introduction

Hypothyroidism is an endocrine disorder characterized by inadequate thyroid hormone production. It may be primary (due to inadequate thyroid hormone production from the thyroid gland itself), secondary (due to inadequate thyroid-stimulating hormone (TSH) production from the pituitary gland), or tertiary (due to inadequate production of thyrotropin-releasing hormone from the hypothalamus). Primary hypothyroidism is defined by a high TSH level and a low free thyroxine level (also known as T4), whereas subclinical hypothyroidism is defined as an elevated TSH level with a normal free thyroxin level. Subclinical hypothyroidism is usually asymptomatic or has milder symptoms as compared to overt hypothyroidism [1,2]. Subclinical hypothyroidism is also associated with a risk of progression to overt hypothyroidism, especially in those with positive anti-thyroid peroxidase antibodies [3]. Clinical hypothyroidism is thought to affect 0.3-0.4% of the general population in the United States, with an estimated prevalence being higher in those older than 60 [4]. Other estimates of the prevalence in Europe and the United States vary between 2% and 5%, with the prevalence in lower-income areas being less understood and thought to vary depending on the prevalence of iodine deficiency in the country or region [4]. The most common cause of hypothyroidism in the United States is Hashimoto's disease, an autoimmune destruction of the thyroid gland. In lower-income countries, iodine deficiency is a leading cause [5]. Subclinical hypothyroidism is thought to be present in 10% of the adult population, with a higher prevalence in older adults [1]. Hypothyroidism most commonly presents with symptoms of cold intolerance, fatigue, weight gain, lethargy, dry skin, constipation, and voice changes [4,5]. Pericardial and pleural effusions are less common manifestations of hypothyroidism, especially if they present in isolation. The incidence of pericardial effusion in hypothyroidism is unknown, and estimates have varied widely between 3% and 37% [6]. The patient in this case report developed primary hypothyroidism in 2014 when he underwent a complete thyroidectomy with external beam radiation therapy due to thyroid cancer. He was subsequently placed on levothyroxine 150 micrograms daily, which adequately replaced his thyroid hormone levels and maintained TSH levels within the normal range. However, he became non-adherent to levothyroxine sometime in 2022, which subsequently led to the development of an isolated and symptomatic pericardial and pleural effusion related to hypothyroidism.

## Case presentation

The patient is a 68-year-old man with a history of hemiparesis affecting the right side after a stroke, alcoholic polyneuropathy, alcohol dependence in sustained remission, major depressive disorder in remission, emphysema, hypertension associated with diabetes, and a history of deep venous thrombosis in the right lower extremity. He presented to the primary care clinic for a follow-up visit on December 16, 2022. During the visit, he had cough, congestion, wheezing, and shortness of breath with exertion that were new in onset. He denied any fevers, chills, or palpitations. He had a normal cardiopulmonary exam. He was given supportive care instructions for acute bronchitis. He returned to the clinic two weeks later with worsening symptoms, including continued coughing with brown sputum and worsening shortness of breath. A chest X-ray interpreted by a radiologist and the research team showed a left para-hilar infiltrate, cardiomegaly, and emphysema. He was started on azithromycin and systemic steroids for presumed pneumonia. Earlier in the month, he had also developed new-onset atrial fibrillation, which was incidentally seen on an electrocardiograph (EKG) in the pre-operative setting for a urologic procedure.

**Figure 1:**
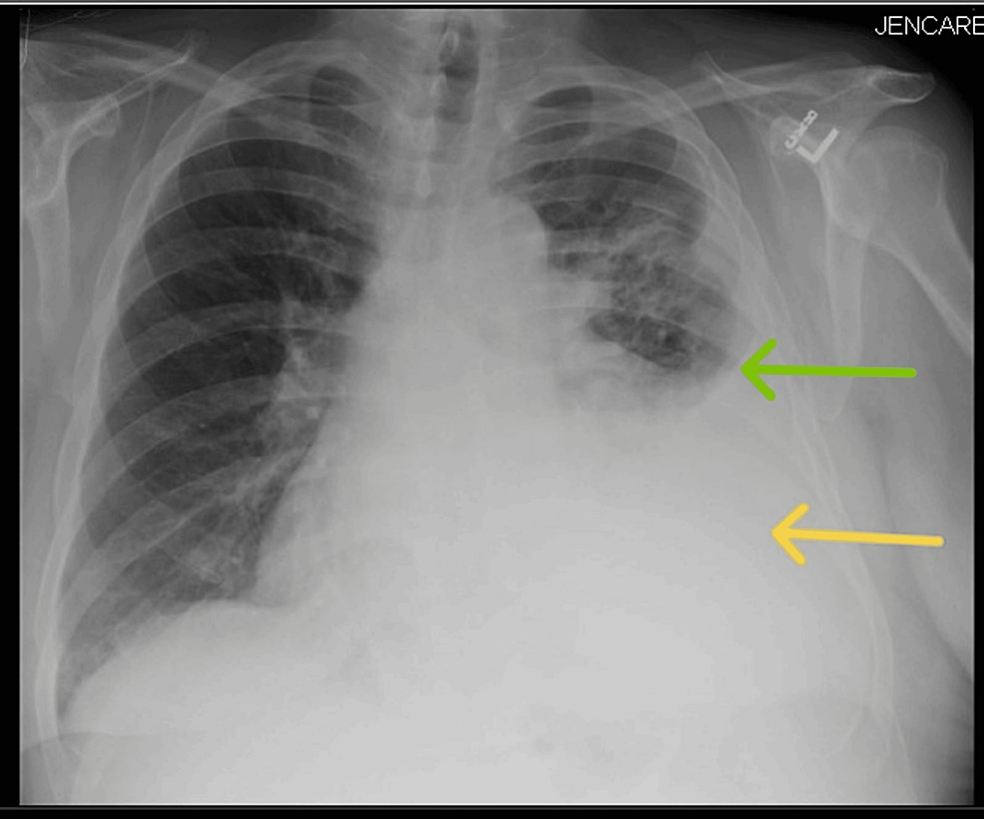
Antero-posterior view chest radiograph showing the large left-sided pleural effusion (yellow arrow) with a Meniscus sign (green arrow)

**Figure 2:**
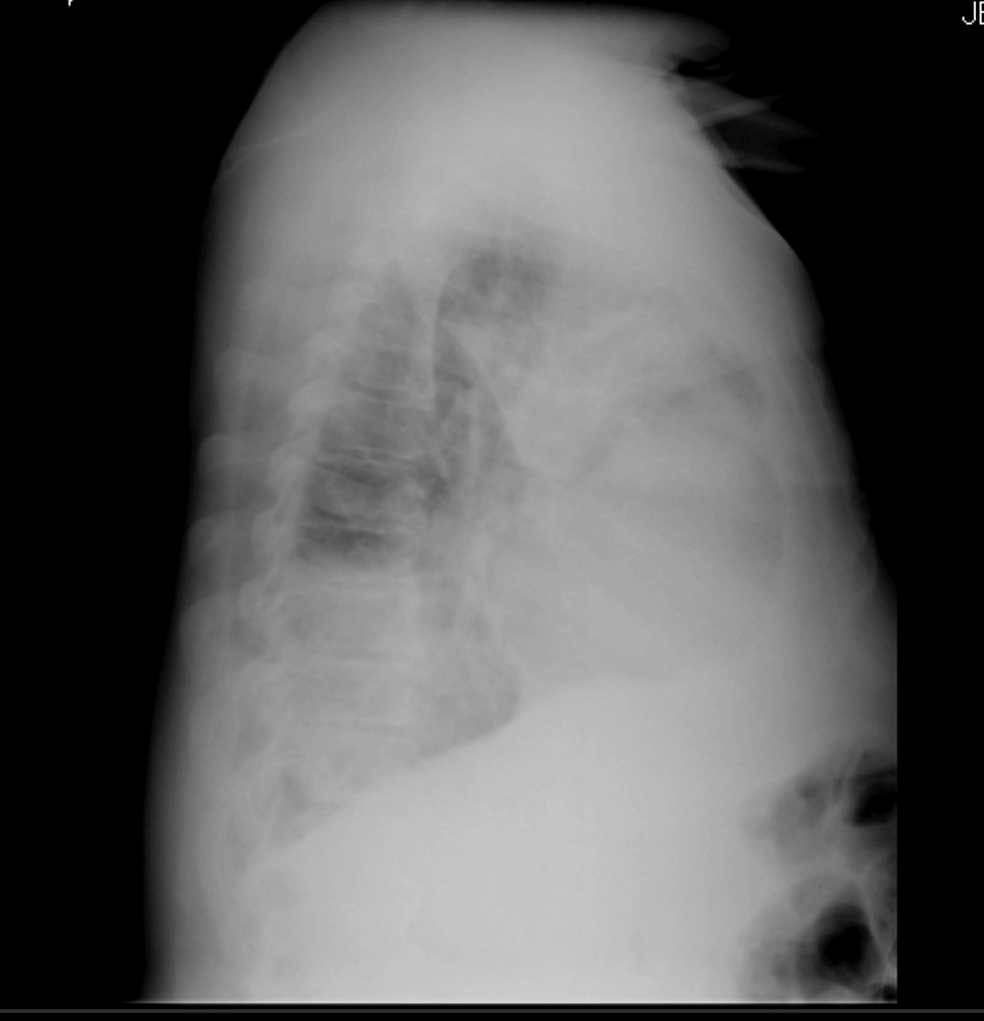
Lateral view chest radiograph showing the pleural effusion

**Figure 3:**
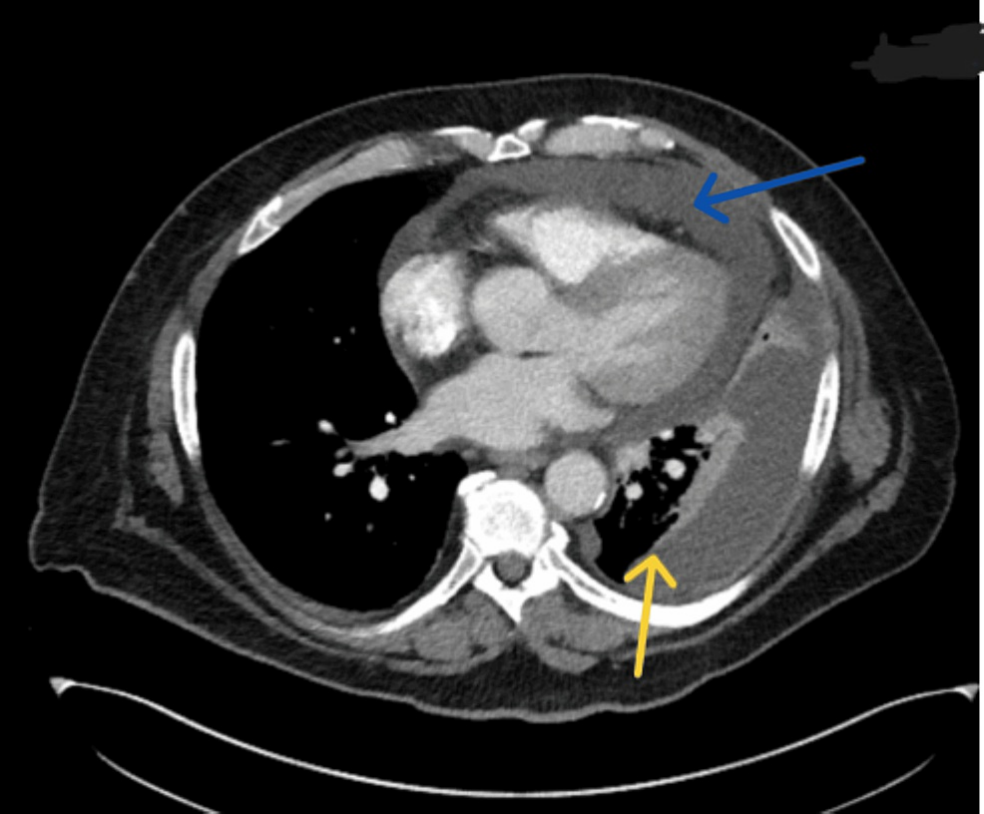
A transverse view CT scan of the chest with contrast from January 2023 showing a large left-sided pleural effusion (yellow arrow) and a pericardial effusion (blue arrow) CT: computed tomography

**Figure 4:**
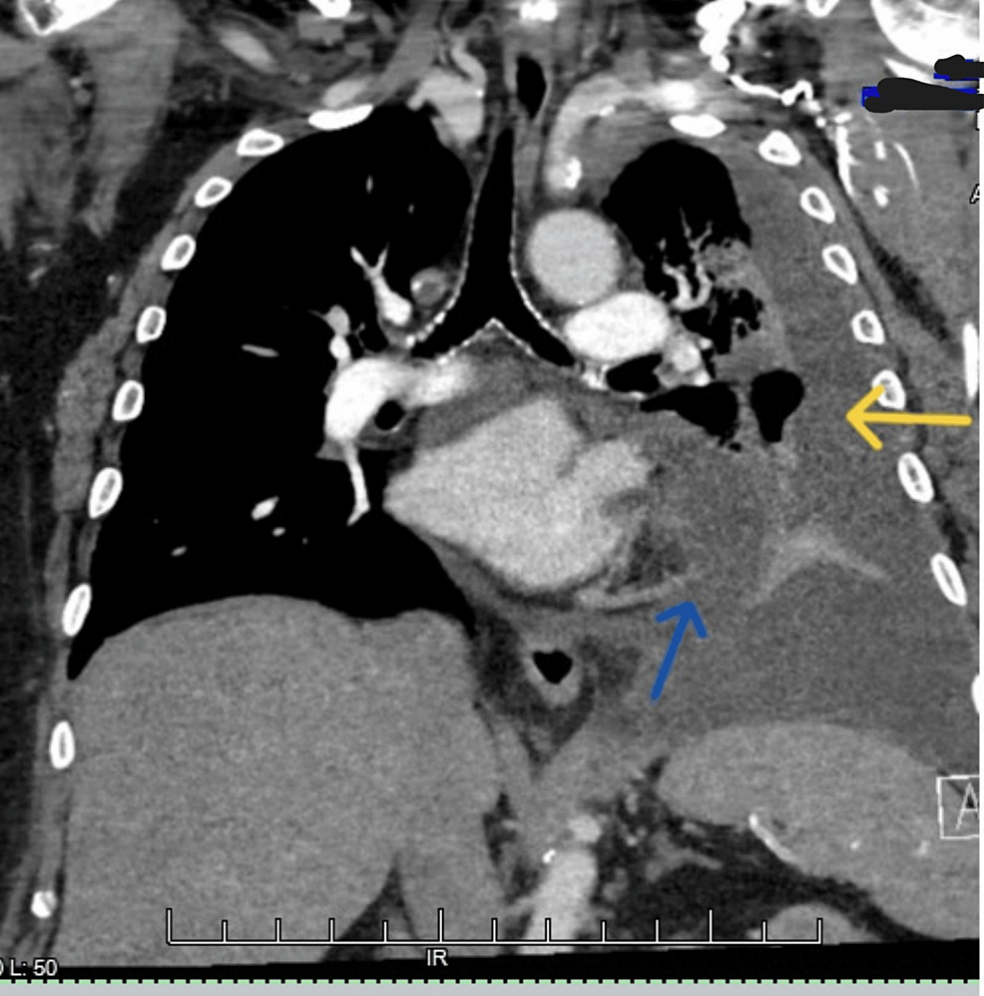
Coronal view CT scan of the chest with contrast from January 2023 showing a large left-sided pleural effusion with loculations (yellow arrow) and a pericardial effusion (blue arrow)

Laboratory testing showed a TSH of 121 mIU/L and a free T4 of 1.4 micrograms per dL (normal laboratory values for TSH are 0.5 to 5.0 mIU/L and for T4 are 5.0 to 12.0 micrograms per dL; Table 1). An EKG showed sinus bradycardia with a rate of 46 with low voltage in the precordial leads and an incomplete right bundle branch block. The patient admitted to being non-adherent to levothyroxine. The patient returned to the office on January 12, 2023, completing the antibiotics and steroids and confirming adherence with levothyroxine. However, he had continued coughing and sputum production. He was counseled on using an expectorant with codeine for symptomatic relief. A repeat chest X-ray on January 26, 2023, showed an increasing left lung consolidation with a large left-sided pleural effusion and left hilar/para-hilar densities, which had progressed from the prior exam as interpreted by a radiologist and the research team (Figures 1-2). A CT chest on the same date showed a moderate pericardial effusion, 2.1 cm in width, and a moderate left pleural effusion partially loculated and associated with consolidation and atelectasis as interpreted by the radiologist (Figures 3-4). There were also right lower lobe and subpleural reticular densities with patchy left upper lobe densities with air bronchograms.

**Table 1:** TSH and thyroxine (free T4) levels The table lists thyroid hormone levels, including TSH and free T4, with corresponding normal ranges since the discovery of severe hypothyroidism on December 16, 2022. The patient resumed his levothyroxine shortly after December 16, 2022. TSH: thyroid-stimulating hormone

	TSH (all values in mIU/L)	Free T4 (all values in mcg/dL)
Normal range	0.5-5.0	5.0-12.0
Date		
December 16, 2022	121	1.4
January 26, 2023	27.3	7.4
February 24, 2023	9.3	1.54

**Figure 5:**
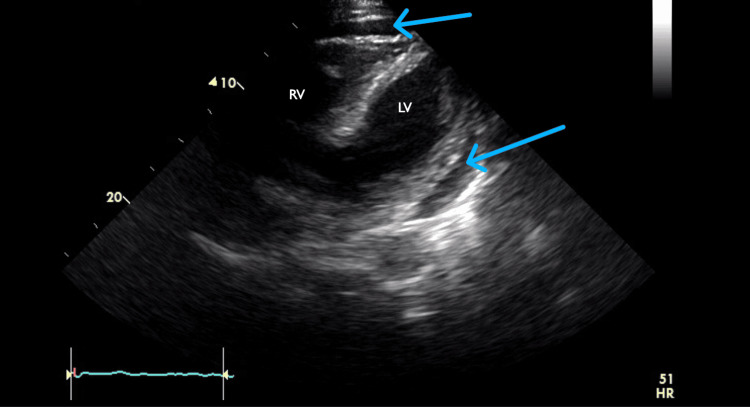
Subcostal echocardiograph view from February 2023 showing the pericardial effusion (blue arrows) with the right ventricle (RV) and left ventricle (LV)

**Figure 6:**
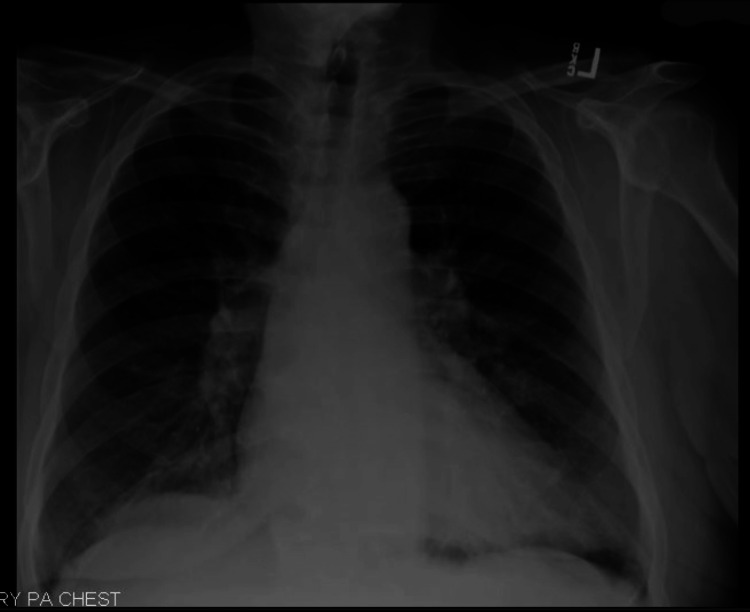
Posterior-anterior chest radiograph from May 2023 showing interval resolution of the pleural effusion

**Figure 7:**
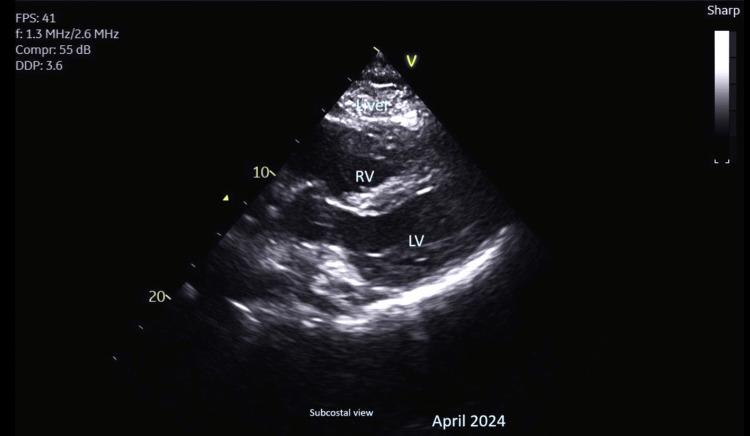
Subcostal echocardiograph view from April 2024 showing resolution of the pericardial effusion with the right ventricle (RV) and left ventricle (LV)

On February 8, 2023, the patient underwent a thoracentesis of the left pleural effusion with 1200 mL of a thin, yellow, clear fluid removed. The analysis of the pleural fluid did not show malignant cells, with only a few reactive lymphocytes and a few mesothelial cells. The pleural fluid analysis also showed 5% neutrophils, 74% lymphocytes, 18% macrophages, and 3% mesothelial cells with an acid-fast bacilli smear, fungal cultures, and gram staining all showing no growth of organisms. The pleural fluid had a total protein of 4.5 grams, glucose of 135 mg/dL, and LDH of 160 U/L. With a serum protein level of 7.0 mg/dL, the pleural fluid protein to the serum protein level of 0.64 was consistent with an exudative effusion based on Light's criteria. An echocardiograph from February 2, 2023, showed a moderate amount of circumferential pericardial effusion with no evidence of tamponade, a left ventricular ejection fraction of 55-65%, and no evidence of diastolic dysfunction (Figure 5). On January 26, 2023, the patient's TSH improved to 27.3 mIU/L with a normalized T4 level of 7.4 micrograms per dL, while he reported adherence to his levothyroxine. His symptoms of coughing, shortness of breath, and sputum production had all been resolved. On February 25, 2023, the TSH was mildly high at 9.3 mIU/L with a free T4 of 1.54 micrograms per dL while the patient remained asymptomatic, and on October 6, 2023, the TSH had normalized to 2.16 mIU/L with a normal free T4 of 1.51 micrograms per dL. A chest X-ray on May 12, 2023, showed complete resolution of the pleural effusion (Figure 6). An echocardiograph was completed in August 2023, which showed normal left ventricular systolic function and a mild concentric pericardial effusion with a moderate appearance only in the subcostal view. The patient saw an interventional cardiologist in October 2023 who stated that the circumferential pericardial effusion was moderate only on the subcostal view (and mild on all other views) and that there was no evidence of tamponade on the echocardiograph and that a pericardiocentesis was not indicated. The patient was not experiencing shortness of breath, chest pain, or other symptoms related to the pericardial effusion at the time. An additional echocardiograph on April 23, 2024, showed a small circumferential pericardial effusion (much smaller than previously), which was not visualized on the subcostal view (it had resolved on this view), and the patient was still asymptomatic, with the previously described symptoms of dyspnea, exercise intolerance, and cough being completely resolved (Figure 7).

## Discussion

This case highlighted an uncommon presentation of primary hypothyroidism, one in which an isolated pericardial and pleural effusion were present without other commonly presenting symptoms seen in profound hypothyroidism such as constipation, cold intolerance, weight gain, dry skin, mood changes, or fatigue [5,7]. The patient did have a history of depression but did not have symptoms of depressive disorder during the hypothyroid period. Symptoms of hypothyroidism are often non-specific and difficult to differentiate from other disorders, often being commonly seen in those who are euthyroid [8]. Thus, a high index of suspicion is required to diagnose clinical or subclinical hypothyroidism with atypical symptoms. The patient in this case presentation had non-specific symptoms of cough, dyspnea, and sputum production related to the pleural and pericardial effusions without any signs or symptoms specific to the common presentation of hypothyroidism. Dyspnea, cough, and pleuritic chest pain are common symptoms of pericardial and pleural effusions in hypothyroidism [2,9]. Pericardial effusions are more common in those with severe hypothyroidism, with most cases being seen in those with TSH levels above 30 mIU/mL in primary hypothyroidism [2]. In addition to the findings of effusions on imaging, the patient did have findings on the EKG suggestive of hypothyroidism and pericardial effusion, including the low voltage of the QRS complexes, sinus bradycardia, and prolongation of the QTc interval (which is a risk factor for ventricular arrhythmias, including Torsade de Pointes) [10]. Bradycardia, a shortened or prolonged QTc interval (with age-specific differences in the interval seen; with a shortened interval in younger women but prolonged in older men), a longer P wave interval, and low-voltage QRS complexes are EKG changes that are specific to hypothyroidism [11].

The pleural effusion in this patient was analyzed and shown to be exudative in character; however, pleural effusions due to hypothyroidism can be both transudative and exudative [12].

The pericardial effusion in hypothyroidism is thought to be due to increased permeability of the epicardial blood vessels and decreased lymphatic drainage [6,13]. The incidence of pleural effusion in hypothyroidism is unknown, and the pathophysiology is thought to involve an increased rate of trans-capillary escape of albumin into the interstitial space with decreased clearance of albumin from the interstitial space, resulting in increased oncotic pressure and fluid accumulation [14].

Treatment of the underlying hypothyroidism with thyroid hormone replacement therapy usually leads to the resolution of pericardial and pleural effusions, as it did in this case [6,10,15]. However, in cases of cardiac tamponade (which is rare in pericardial effusion associated with hypothyroidism) or hemodynamic instability, a pericardiocentesis or thoracentesis may be needed [6,10].

Patients with severe hypothyroidism are also at high risk of decompensating into myxedema coma, involving multi-organ dysfunction and progressive mental deterioration, which may lead to death with a mortality rate of greater than 50% [16,17]. In this case, the patient did not have any precipitating factors such as illness, infection, trauma, medications, or exposure to a cold, which may have led to a myxedema coma [18]. As such, the case illustrates the importance of considering hypothyroidism in the differential diagnosis of such a presentation.

This case also highlighted the importance of assessing medication adherence in all patients, as many patients with chronic medical conditions are non-adherent to their medications. It is estimated that 30-50% of patients with chronic cardiovascular disease or associated risk factors such as hypertension or hyperlipidemia are not completely adherent to their medications [19]. In patients with hypothyroidism on long-term thyroid replacement therapy, only 36.8% achieved drug adherence rates greater than 80% [20].

## Conclusions

This case report illustrates an uncommon and often overlooked presenting sign of hypothyroidism: pericardial and pleural effusions. This case report demonstrates the importance of considering hypothyroidism as a potential cause of pericardial or pleural effusions, as prompt treatment with thyroid hormone replacement is easily initiated and usually resolves the effusions and associated symptoms. The case also illustrates the variety of clinical signs and symptoms as well as radiologic and EKG findings of effusions in hypothyroidism, which may aid in their prompt recognition and initiation of treatment. The case also highlights the clinical importance of identifying severe hypothyroidism early in order to prevent progression to a rare but lethal myxedema coma. Future research is needed to validate radiographic features and pleural or pericardial fluid indices that are specific to pleural or pericardial effusions in hypothyroidism. Research may also aid in identifying patients who are hypothyroid and are at risk of developing effusions.
